# Renin-Angiotensin System and Coronavirus Disease 2019: A Narrative Review

**DOI:** 10.3389/fcvm.2020.00143

**Published:** 2020-08-11

**Authors:** Annamaria Mascolo, Cristina Scavone, Concetta Rafaniello, Carmen Ferrajolo, Giorgio Racagni, Liberato Berrino, Giuseppe Paolisso, Francesco Rossi, Annalisa Capuano

**Affiliations:** ^1^Section of Pharmacology “L. Donatelli”, Department of Experimental Medicine, University of Campania “Luigi Vanvitelli”, Naples, Italy; ^2^Campania Regional Centre for Pharmacovigilance and Pharmacoepidemiology, Naples, Italy; ^3^Department of Pharmacological and Biomolecular Sciences, Università degli Studi di Milano, Milan, Italy; ^4^Department of Advanced Medical and Surgical Sciences, University of Campania “Luigi Vanvitelli”, Naples, Italy

**Keywords:** COVID-19, renin-angiotensin system, SARS-COV-2, heart damage, pulmonary damage, RAS inhibitors

## Abstract

Although clinical manifestations of the 2019 novel coronavirus disease pandemic (COVID-19), caused by the novel severe acute respiratory syndrome coronavirus 2 (SARS-COV-2), are mainly respiratory symptoms, patients can also develop severe cardiovascular damage. Therefore, understanding the damage caused by SARS-COV-2 to the cardiovascular system and the underlying mechanisms is fundamental. The cardiovascular damage may be related to the imbalance of the renin-angiotensin-system (RAS) as this virus binds the Angiotensin-Converting-Enzyme 2 (ACE2), expressed on the lung alveolar epithelial cells, to enter into cells. Virus internalization may cause a downregulation of ACE2 on host cell surface that could lead to a local increased level of angiotensin II (AII) and a reduced level of angiotensin 1-7 (A1-7). An imbalance between these angiotensins may be responsible for the lung and heart damage. Pharmacological strategies that interfere with the viral attachment to ACE2 (umifenovir and hydroxychloroquine/chloroquine) or that modulate the RAS (analogous of A1-7 and ACE2, losartan) are in clinical development for COVID-19. The use of RAS inhibitors has also become a matter of public concern as these drugs may increase the mRNA expression and levels of ACE2 and impact the virulence and transmission of SARS-COV-2. Data on the effect of RAS inhibitors on ACE2 mRNA expression are scarce. Scientific societies expressed their opinion on continuing the therapy with RAS inhibitors in patients with COVID-19 and underlying cardiovascular diseases. In conclusion, RAS may play a role in SARS-COV-2-induced cardiac and pulmonary damage. Further studies are needed to better understand the role of RAS in COVID-19 and to guide decision on the use of RAS inhibitors.

## Introduction

The renin–angiotensin system (RAS) is a complex hormonal system composed by different mediators that can affect the cardiovascular, renal, immune, and nervous functions (1, 2). Many components of the RAS have been isolated from different tissues (3), including the lung (4). This system is composed by two pathways: the classic RAS and the non-classic RAS, which have opposite activities, especially for renal, and cardiovascular functions (2, 5). A component of the non-classic RAS, the Angiotensin-Converting-Enzyme 2 (ACE2) present on the lung surface, has been discovered to be a functional receptor for coronaviruses, essential for triggering their infection (1). Severe acute respiratory syndrome coronavirus 1 (SARS-COV-1) and SARS-COV-2, which are responsible for the SARS and the more recent coronavirus disease 2019 (COVID-19), respectively, are both able to bind the ACE2 in the lung (6, 7). Patients affected with COVID-19 show respiratory and flu-like symptoms, which can be complicated by lymphopenia and interstitial pneumonia with high levels of pro-inflammatory cytokines that can lead to acute respiratory distress syndrome (ARDS) and organ failure (8). Although the clinical manifestations of COVID-19 are mainly represented by respiratory symptoms, some patients also developed severe cardiovascular damage (9). In addition, an increased risk of death was found in patients with cardiovascular diseases (9).

Understanding the mechanisms by which the RAS interacts with SARS-COV-2 is fundamental for the treatment of patients with cardiac diseases as showed in the context of metabolic diseases (10). Moreover, considering the interaction between these viruses and the ACE2, concerns were also raised about the use of RAS inhibitors in patients with COVID-19 as they may alter ACE2 mRNA expression and levels and, in this way, impact the virulence and transmission of SARS-COV-2 (11). Therefore, in this review, we aim to summarize the physiological role of the RAS, its implication in the SARS-COV-2 infection, the actual evidence and recommendation on the use of RAS inhibitors, and the ongoing researches of drugs with a potential for the treatment of COVID-19 and acting either by influencing the RAS or disrupting the viral attachment to ACE2.

## Clinical Characteristics of COVID-19

First evidence regarding the clinical characteristics of patients with COVID-19 showed the presence of bilateral lung ground glass opacity on computed tomography (CT) imaging (12). CT abnormalities were observed in both asymptomatic or symptomatic patients with SARS-CoV-2 infection, making it a useful diagnostic tool. Asymptomatic individuals with CT abnormalities rarely developed severe pneumonia (13). Initial symptoms were fever, cough, dyspnea, myalgia or fatigue, sputum production, headache, hemoptysis, and diarrhea. In most severe cases, there was a progression to ARDS, to acute cardiac injury, to acute kidney injury (AKI), or to shock. Other symptoms that were identified pertained to the gastrointestinal system (nausea and diarrhea) (12). However, other studies showed a lower development of gastrointestinal symptoms (14, 15). Moreover, an increase in serum lactate dehydrogenase as marker of lung tissue damage was observed in COVID-19 patients (13), and it was associated with higher odds of severe disease (14). Additionally, older age and lymphopenia were identified as potential risk factors for severe COVID-19 (13).

## Classic and Non-classic RAS

**Figure 1:**
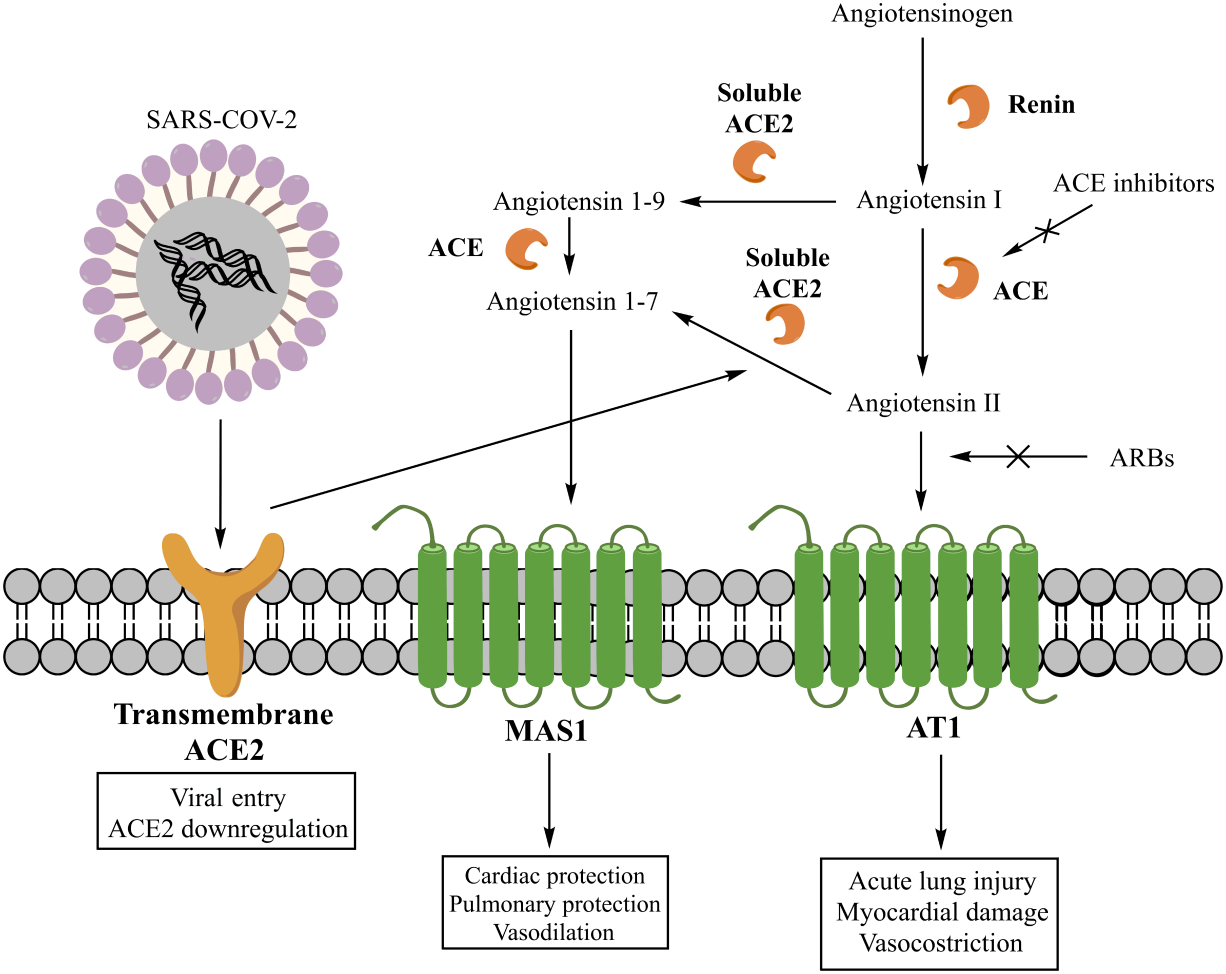
Classic and non-classic renin-angiotensin system (RAS) and its interaction with SARS-COV-2.

The classic RAS involves as main effector peptide the angiotensin II (AII), whose synthesis starts with the cleavage of angiotensinogen into angiotensin I (AI) by the renin and then its conversion into AII by the ACE (16) (Figure 1). Despite this represent the main pathway for the AII production, also other enzymes can be involved (5). The main effects of AII are explained by its interaction with three receptors (AT1, AT2, and nonAT1nonAT2). AT1 and AT2 are classified as G protein-coupled receptors (16), while nonAT1nonAT2 seems more prone to be an angiotensin clearance receptor or an angiotensinase (17). The stimulation of the AT1 receptor can induce vasoconstriction, increase the release of catecholamines and the synthesis of aldosterone (16). Moreover, AT1 receptors can stimulate fibrosis, inflammatory processes, reduction of collagenase activity, and expression of mitogen-activated protein kinase (MAPK) (2, 5). As pro-inflammatory action, these receptors seem to be involved in several pathways: down-regulation of the NADPH oxidase expression in smooth muscle cells; enhancement of the production of reactive oxygen species (ROS) and the activity of pro-inflammatory transcription nuclear factors like nuclear factor-kappaB (NF-kB) and E26 transformation-specific sequence (Ets) (18); release of different types of cytokines such as TNF-α, IL-6, and MCP-1 (19); shifting of the macrophage phenotype toward the pro-inflammatory M1 polarization state (20). The stimulation of AT2 receptors, instead, has a protective role in the RAS activation inducing anti-inflammatory, anti-oxidative, and anti-fibrotic effects (16).

The non-classic RAS involves, instead, other peptide mediators and enzymes. Specifically, the main mediator is the angiotensin 1-7 (A1-7), whose synthesis can involve two different pathways. One starts with the cleavage of AII into A1-7 by the carboxypeptidase ACE2, while another through the cleavage of AI into angiotensin 1–9 (A1–9) by ACE2 and its subsequent conversion into A1–7 by ACE (5) (Figure 1). Today, two forms of ACE2 are recognized, one soluble and another transmembrane, both contributing to the generation of A1-7. The A1–7 stimulates the G protein-coupled receptor MAS1, promoting the nitric oxide release (21), Akt phosphorylation (22), and anti-inflammatory effects (23). Moreover, the activation of MAS1 receptors, expressed on the macrophage surface, inhibits the inflammatory macrophage phenotype and the release of pro-inflammatory cytokines (5). Therefore, A1-7 is a component of a beneficial axis of the RAS that exerts opposite cardiovascular and renal effects compared to the ACE/AII/AT1 axis (24).

Interestingly, it has been found that human monocytes can express ACE and ACE2 and metabolize AI to multiple angiotensin peptides. In particular, classical monocytes (CD14^++^CD16^−^) produce both AII and A1–9/A1–7, whereas the non-classical subtype (CD14^+^CD16^++^) produces mainly A1–7 (25). This indicates that ACE and ACE2 participate to the inflammation also as components of a local RAS at sites infiltrated by monocytes/macrophages.

## SARS-COV-2 and ACE2 in the Lung

SARS-COV-2 is a betacoronavirus with a single-stranded positive-sense RNA genome encapsulated within a membrane envelope (26). The genome encodes for several structural proteins, including the glycosylated spike (S) protein that is a major inducer of host immune response. The S protein is also important because mediates host cell invasion by SARS-COV-2 via binding to the receptor protein ACE2 present on the surface of lung alveolar epithelial cells (host cells) (6, 27). The affinity of S protein binding region to the extracellular domain of ACE2 has been estimated of 15 nM (27, 28). The invasion process requires the activation of the S protein, which is facilitated by the human androgen-sensitive transmembrane serine protease type 2 (TMPRSS211) (6, 26). Specifically, TMPRSS211 cleaves the S protein and generates the S1 and S2 subunits. This is a critical step as both subunits are essential for viral entry in the host cells (28). S1 is the subunit recognized by ACE2 and the one that facilitates viral attachment, whereas S2 is the subunit that drives membrane fusion and viral internalization in the pulmonary epithelium (6). The greater virulence of SARS-COV-2 compared to SARS-COV-1 was supposed to be related to the higher affinity of S1 subunit for ACE2 (26, 28). In fact, a Cryo-EM structure analysis revealed that the affinity of the S protein of SARS-COV-2 to ACE2 is about 10–20 times greater than that observed with the S protein of SARS-COV-1 (27).

Another important consideration is that the ACE2 internalization mediated by SARS-COV-2 could potentially result in a reduced presence of ACE2 on cell surface, leading to the absence of a key factor for AII degradation and A1-7 synthesis. An imbalance between AII and A1-7 levels may further exacerbate the damage of lung provoked by SARS-COV-2. Therefore, a decrease in ACE2 may contribute to the reduction of pulmonary function and the increase of tissue fibrosis and inflammation due to COVID-19 (28). This hypothesis was already investigated with SARS-COV-1 infection, which was associated with a reduced presence of ACE2 on cell membranes and an increased severity of lung injury (29). Because SARS-COV-1 and SARS-COV-2 share the same cellular invasion process, they may also share similar pathogenesis and pathological manifestations of lung injury (29).

## SARS-COV-2 and ACE2 in the Heart

Potentially, once the SARS-COV-2 enters the circulation, it can infect any tissue expressing the ACE2, including the heart or other cardiovascular tissues (28). Evidence showed that patients with COVID-19 had a high occurrence of cardiovascular symptoms, in addition to respiratory ones, and that these symptoms were also reported in patients without underlying cardiovascular diseases (30). The National Health Commission of China (NHC) reported that cardiovascular symptoms (such as heart palpitations and chest tightness) occurred at the beginning of the SARS-COV-2 infection in some of confirmed cases. Moreover, the 11.8% of patients who died for COVID-19 but without underlying cardiovascular diseases had substantial heart damage (30). These data suggest the necessity of involving cardiologists in the management of patients with COVID-19 (31). However, the real contribute of SARS-COV-2 in the development of myocardial injury is not clear (32). It is known that the infection itself may directly impact cardiovascular diseases and the development of cardiovascular complications (30, 33). Another factor that should be considered is also the expression in the tissue of TMPRSS211 or other proteases able to trigger the viral entry (6). Another hypothesis for the induction of heart damage considers the reduction of ACE2 caused by SARS-COV-2, which might exacerbate symptoms in patients with underlying cardiovascular diseases (28, 34). This could be due to the imbalance between the classic and non-classic RAS in favor of AII that may further compromise cardiac function apart from the viral infection (28). In fact, a preclinical study shows that ACE2 knockout animal models had a worse left ventricular remodeling in response to the AII-induced acute injury, suggesting a protective role of non-classic RAS in myocardial recovery (35). This finding may also explain the heart damage found in patients with COVID-19 but without cardiovascular diseases (30). To corroborate this hypothesis, a study demonstrated that the AII level in the plasma sample of SARS-COV-2 infected patients was markedly high and linearly associated with the viral load and lung injury (32). Moreover, another study found in the 35% of heart samples from patients with SARS the presence of viral RNA associated with a reduced ACE2 protein expression (36). Another proposed mechanism of myocardial injury includes the cytokine storm (32) as the systemic inflammatory response and immune system disorders during disease progression may be responsible for the myocardial damage (30). Also, in this case, other than the viral infection itself, a minor role in potentiating the inflammation might be played by the classic RAS cascade. Moreover, needs to be considered that also some drugs that are being investigated for COVID-19 are potential risk factors for the cardiovascular toxicity (31).

Finally, evidence showed that COVID-19 may produce a form of disseminated intravascular coagulation (DIC) as the presence of microthrombi have been reported from the autopsy of patients with COVID-19 (37). To date, the exact causes of DIC are many and unclear. Potential suggested mechanisms are as follows: inflammation (e.g., IL-6) stimulates the synthesis of fibrinogen (38); or the virus may directly bind to endothelial cells; or a mutual relationship between DIC and cytokine storm (wherein each exacerbates the other) exists.

## Concerns, Evidence and Recommendation on the Use of RAS Inhibitors in Patients With COVID-19

Concerns were raised on the use of RAS inhibitors in patients with COVID-19 as the use of these drugs may determine an increase of ACE2 and then of SARS-COV-2 virulence (11, 30). Among drugs able to inhibit the RAS, there are renin inhibitors, ACE inhibitors, and the Angiotensin Receptor Blockers (ARBs). ACE inhibitors and ARBs are among drugs most commonly used worldwide for the treatment of cardiovascular diseases. Therefore, concerns on their use in patients with COVID-19 are even more important. Initial evidence showed that patients with COVID-19 and coexisting cardiovascular conditions had a more severe illness, a more frequent admission to the intensive care unit, were more prone to receive mechanical ventilation, or to die (11). The first hypothesis was that the medical management of these conditions, including the use of RAS inhibitors, may have contributed to the adverse health outcomes. So far, there is no rigorous report accounting for key factors as potential confounders in risk prediction; moreover, available evidence on the effect of RAS inhibitors on ACE2 mRNA expression and levels are conflicting and scarce, highlighting also the absence of data on lung-specific mRNA expression of ACE2 (11). Researches have also suggested that this effect of RAS inhibitors may not be uniform among molecules (11, 39). Moreover, even if there was a relationship between the RAS inhibition and the up-regulation of ACE2, there is no evidence demonstrating a causal relationship between the ACE2 activity and the SARS-COV-2 associated mortality (40). Furthermore, the presence of ACE2 on cell surface may not be the only factor participating in the infection process. In fact, additional co-factors might participate in the cell invasion process as SARS-COV-1 infection was not observed in some cells expressing ACE2 on the surface, whereas it was found in cells apparently without ACE2 (41). Moreover, the lethal outcome observed in patients with COVID-19 may also be driven by the severity of the lung damage. In this regard, a preclinical study suggested a beneficial role of RAS blockers in limiting the SARS-COV-1-induced lung injury (42), so that, a protective role is played by RAS inhibitors. This finding could rise a new hypothesis in which the activation of the classic RAS, rather than its inhibition, may predispose patients toward a more deleterious outcome.

Finally, another aspect that should be considered is the potential harm associated with the withdrawal of a RAS inhibitor in a patient with a stable cardiovascular condition. In fact, RAS inhibitors are known to determine clinical benefits and to protect both myocardium and kidney. Therefore, their sudden withdrawal may expose patients to an unjustified risk related to decompensation and symptoms exacerbation, especially in high cardiovascular risk patients. In this regard, clinical trials have demonstrated a rapid relapse of the dilated cardiomyopathy or a decline of the clinical condition after the discontinuation of the pharmacological treatment with a RAS inhibitor (43).

Moreover, there are solid evidence on the effect of RAS inhibitors in reducing mortality in patients with cardiovascular diseases. These drugs are indeed the cornerstone therapy for a favorable prognosis in patients with heart failure, with the highest level of evidence in reducing mortality (44). Finally, Scientific Societies have expressed their opinion on the use of RAS inhibitors, highlighting the absence of evidence suggesting an eventual discontinuation of ACE-inhibitors, or ARBs in patients with COVID-19. Therefore, they recommend to continue the treatment with the usual anti-hypertensive agent in patients with COVID-19 (45–49). This recommendation has been supported by different observational studies published in the last few months. In this regards, a population-based case–control study carried out in the Lombardy region of Italy did not show any association between the use of ARBs or ACE-inhibitors with COVID-19 among all patients (adjusted odds ratio, 0.95 [95% confidence interval (CI), 0.86 to 1.05] for ARBs and 0.96 [95% CI, 0.87 to 1.07] for ACE inhibitors) or among patients with a severe or fatal course of the disease (adjusted odds ratio, 0.83 [95% CI, 0.63 to 1.10] for ARBs and 0.91 [95% CI, 0.69 to 1.21] for ACE inhibitors) (50).

Accordingly, another Italian nested case-control study showed no increased risk of being infected by SARS-COV-2 in patients treated with RAS inhibitors (51). Moreover, a case-population study showed that RAS inhibitors had an adjusted odds ratio for COVID-19 requiring admission to hospital of 0.94 (95% CI, 0.77 to 1.15) compared with users of other antihypertensive drugs (52). In relation to the mortality outcome, instead, a retrospective observational study showed similar mortality rates between the RAS inhibitor and non-RAS inhibitor cohorts (2.2 vs. 3.6%, adjusted hazard ratio [HR] 0.85; 95% CI, 0.28 to 2.58) (53). Similarly, a Korean nationwide population-based cohort study showed no difference for mortality between RAS inhibitors users and non-users (adjusted odds ratio, 0.88; 95% CI, 0.53 to 1.44) (54). Finally, a retrospective, multi-center study demonstrated a lower risk of COVID-19 mortality in inhospital patients with hypertension and hospitalized due to COVID-19 who received ACE inhibitor/ARB compared to those who did not receive an ACE inhibitor/ARB (adjusted HR, 0.37; 95% CI, 0.15 to 0.89) (55). Different other published studies supported the aforementioned findings (56–58). Moreover, it is ongoing an observational study that will enroll about 2,000 participants to assess if the chronic intake of RAS inhibitors modifies the prevalence and severity of clinical manifestations of COVID-19 (ClinicalTrials.gov identifier, NCT04331574).

Clinical trials are also ongoing to assess instead clinical benefits of continuing or not the treatment with ARBs or ACE inhibitors in patients with COVID-19 (NCT04330300, NCT04351581, NCT04353596, and NCT04329195). In particular, the NCT04330300 is a randomized, open label, parallel assignment clinical trial that will randomize patients with primary essential hypertension who are already taking ACE inhibitor/ARB to either switch to an alternative antihypertensive agent or continue with the ACE inhibitor/ARB treatment. The NCT04351581 is a randomized, single mask (outcome assessor), parallel assignment clinical trial that will randomize hospitalized patients with COVID-19 to continue or discontinue their treatment with the ACE inhibitor or ARB. The NCT04353596 is also a randomized, single mask (outcome assessor), parallel assignment clinical trial that will randomize symptomatic SARS-CoV2-infected patients to stop/replace the chronic treatment with the ACE inhibitor/ARB or to continue this chronic treatment. The NCT04329195 is instead a randomized, open label, parallel assignment clinical trial that will randomize patients with a history of cardiovascular disease treated with RAS blockers, and infected by SARS-CoV-2 to stop or continue the treatment with the RAS blocker. Moreover, the substudy of the Austrian Coronavirus Adaptive Clinical Trial (ACOVACT), which is a randomized, controlled, multicenter, open-label basket trial that aims to compare various antiviral treatments for COVID-19, will also compare the sub-arm with RAS blockade vs. no RAS blockade for patients with blood pressure >120/80 mmHg (NCT04351724). Characteristics of the ongoing clinical trials are showed in Table 1.

**Table 1:** Characteristics of ongoing clinical trials on drugs acting either by influencing the RAS or disrupting the viral attachment to ACE2 in patients with COVID-19.

**Clinical trial number**	**Clinical phase; multicenter**	**Arms**	**Estimated enrollment**	**Primary outcome**	**Estimated study completion date**
NCT04330300	4; No	• Experimental arm: switching to an alternative anti-hypertensive medication (specifically a calcium channel blocker or thiazide/thiazide-like diuretic at an equipotent blood pressure lowering dose). The choice of the alternative anti-hypertensive will be at the discretion of the patient's treating physician. • Comparator arm: continuing the treatment with ACE inhibitor/ARB	2,414	1. Number of COVID-19 positive participants who die, require intubation in intensive care unit, or require hospitalization for non-invasive ventilation at 12 months. Time from randomization to the first occurrence of any of the clinical events above.	March 1, 2021
NCT04351581	Not reported; No	• Experimental arm: continuing the treatment with ACE inhibitor/ARB. The clinicians will be encouraged to continue the medication throughout the hospital admission but it will be permissible for the clinician to stop treatment if necessary (e.g., due to hypotension). • Experimental arm: discontinuing the treatment with ACE inhibitor/ARB. If hypertensive treatment is necessary during hospital admission, the clinicians will first be encouraged to start non-ACE inhibitor/non-ARB treatment.	215	1. Days alive and out of hospital within 14 days after recruitment	December 2020
NCT04353596	4; Yes	• Experimental arm: chronic treatment with ACE inhibitor or ARB will be stopped or replaced. • Comparator arm: no intervention, which means to continue the treatment with ACE inhibitor or ARB.	208	1. Combination of maximum Sequential Organ Failure Assessment (SOFA) Score and death at 30 days. 2. Composite of admission to an intensive care unit, the use of mechanical ventilation, or all-cause death at 30 days.	May 15, 2022
NCT04329195	3; No	• Experimental arm: discontinuation of RAS blocker therapy • Comparator arm: continuation of RAS blocker therapy	554	1. Time to clinical improvement from day 0 to day 28 (improvement of two points on a seven-category ordinal scale, or live discharge from the hospital, whichever comes first)	August 9, 2020
NCT04351724substudy	2/3; Yes	• Experimental arm: candesartan at 4 mg once daily and titrated to normotension • Comparator arm: non-RAS antihypertensive agents titrated to normotension. Those with normal blood pressure may be controlled without further treatment.	500	1. Sustained improvement (>48 h) of one point on the WHO Scale within 29 days (daily evaluation).	December 31, 2020
NCT04260594	4; Not reported	• Experimental arm: umifenovir tablets (2 tablets/time, 3 times/day for 14–20 days) + basic treatment • Comparator arm: basic treatment • The basic treatment is based on the condition of the patient.	380	1. Virus negative conversion rate in the first week	December 30, 2020
NCT04252885	4; No	• Experimental arm: standard treatment + lopinavir/ritonavir. Specifically, 50 participants are given ordinary treatment plus a regimen of lopinavir (200 mg) and ritonavir (50 mg) (oral, q12h, every time 2 tablets of each, taking for 7–14 days).	125	1. The rate of virus inhibition at Day 0, 2, 4, 7, 10, 14, and 21. Novel corona viral nucleic acid is measured in nose/throat swab at each time point.	July 31, 2020
		• Comparator arm: standard treatment + umifenovir. Specifically, 50 participants are given ordinary treatment plus a regimen of umifenovir (100 mg) (oral, tid, 200 mg each time, taking for 7–14 days). • No intervention arm: standard treatment. Specifically, 25 cases are only given ordinary treatment.			
NCT04255017	4; No	• Experimental arm: addition of umifenovir (0.2 g once, 3 times a day for 2 weeks) • Experimental arm: addition of oseltamivir (75 mg once, twice a day for 2 weeks) • Experimental arm: addition of lopinavir/ritonavir (500 mg once, twice a day for 2 weeks) • No intervention arm: symptomatic supportive treatment	400	1. Rate of disease remission at 2 weeks. Defined for mild patients as fever, cough and other symptoms relieved with improved lung CT, and for severe patients as fever, cough and other symptoms relieved with improved lung CT, SPO2> 93% or PaO2/FiO2 > 300 mmHg (1 mmHg = 0.133 Kpa); 2. Time for lung recovery at 2 weeks. Defined as the comparison of the average time of lung imaging recovery after 2 weeks of treatment in each group.	July 1, 2020
NCT04350684	4; No	• Experimental arm: umifenovir + interferon-β 1a + lopinavir/ritonavir + single dose of hydroxychloroquine + standards of care • Comparator arm: interferon-β 1a + lopinavir/ritonavir + single dose of hydroxychloroquine + standards of care	40	1. Time to clinical improvement from the date of randomization until 14 days later. Improvement of two points on a seven-category ordinal scale (recommended by the World Health Organization: COVID-2019) R&D. Geneva: World Health Organization) or discharge from the hospital, whichever came first.	April 24, 2020
NCT04312009	2; Yes	• Experimental arm: losartan (50 mg daily, oral) • Control arm: placebo (microcrystalline methylcellulose, gelatin capsule, oral)	200	1. Difference in Estimated Positive End-expiratory Pressure (PEEP adjusted) P/F Ratio at 7 days. Outcome calculated from the partial pressure of oxygen or peripheral saturation of oxygen by pulse oximetry divided by the fraction of inspired oxygen (PaO2 or SaO2: FiO2 ratio). PaO2 is preferentially used if available. A correction is applied for endotracheal intubation and/or positive end-expiratory pressure. Patients discharged prior to day 7 will have a home pulse oximeter send home for measurement of the day 7 value, and will be adjusted for home O2 use, if applicable. Patients who died will be applied a penalty with a P/F ratio of 0.	April 1, 2021
NCT04311177	2; Yes	• Experimental arm: losartan (25 mg daily, oral) • Comparator arm: placebo (microcrystalline methylcellulose, gelatin capsule, oral)	580	1. Hospital Admission within 15 days. Outcome reported as the number of participants per arm admitted to inpatient hospital care due to COVID-19-related disease within 15 days of randomization.	April 1, 2021
NCT04328012	2/3; Yes	• Experimental arm: lopinavir/ritonavir (400 mg/200 mg, oral, BID X 5–14 days depending on availability) • Experimental arm: hydroxychloroquine (400 mg BID on Day 0, and 200 mg BID Days 1–4, days 1–13 if available) • Experimental arm: losartan (25 mg, oral, daily X 5–14 days depending on availability) • Comparator arm: placebo (BID X 14 days)	4,000	1. National Institute of Allergy and Infectious Diseases COVID-19 Ordinal Severity Scale (NCOSS) at 60 days. Difference in NCOSS scores between the different treatment groups	April 1, 2021
NCT04335786	4; Yes	• Experimental arm: valsartan for 14 days at a dosage and frequency titrated to blood pressure with 80 mg or 160 mg tablets up to a maximum dose of 160 mg b.i.d. • Comparator arm: placebo for 14 days (matching 80 or 160 mg placebo tablets at a dosage and frequency titrated to systolic blood pressure)	651	1. First occurrence of intensive care unit admission, mechanical ventilation or death within 14 days. Death is defined as all-cause mortality	December 2021
NCT04360551	2; No	• Experimental arm: telmisartan (40 mg, oral, daily X 21 days) • Comparator arm: placebo (once daily X 21 days)	40	1. Maximum clinical severity of disease over the 21 day period of study. Based on a modified World Health Organization (WHO) COVID-19 7-point ordinal scale	June 30, 2021

## New Pharmacological Approaches for Preventing Viral Entry of SARS-COV-2 With a Focus on the Disruption of S Protein/ACE2 Interaction

To prevent viral infection, molecules like camostat mesylate, nafamostat mesylate, gabexate, umifenovir, and hydroxychloroquine/chloroquine are being considered (26). Nafamostat and camostat are inhibitors of the protease TMPRSS211 (26). Gabexate has instead multiple mechanisms of action. It has anticoagulant and anti-platelet activities on one hand, and it is a serine protease inhibitor with antiviral and anti-inflammatory properties on the other (59, 60).

While these drugs act on the protease inhibition, umifenovir and hydroxychloroquine/chloroquine directly influence the S protein/ACE2 interaction (Table 2) (26). Hydroxychloroquine and chloroquine, in addition to their use for malaria and autoimmune diseases, may be effective also for the treatment of COVID-19. These drugs are able to elevate endosomal pH and interfere with ACE2 glycosylation (26, 70). The efficacy of chloroquine was already demonstrated with SARS-COV-1 infection, in which the treatment was effective either if administrated prior or after the infection, suggesting that chloroquine may have both a prophylactic and therapeutic use (70). Moreover, preliminary *in vitro* results demonstrated that remdesivir and chloroquine are highly effective in the inhibition of SARS-COV-2 infection (71). Clinical findings also confirmed the efficacy of chloroquine in terms of reduction of exacerbation of pneumonia and duration of symptoms in a cohort of 100 subjects (72, 73). This finding led the China Authority to include these medicines in the recommendations for the prevention and treatment of COVID-19 pneumonia (73). Many other clinical studies are ongoing to evaluate the efficacy and safety of hydroxychloroquine for the pre-exposure prophylaxis, post-exposure prophylaxis, and treatment of COVID-19 (www.clinicaltrials.gov) (74). However, it should be noted that current evidence on the effects of chloroquine is conflicting. Authors of a recent systematic review underlined that, even though a rationale to justify clinical research on chloroquine in patients with COVID-19 exists, high-quality clinical trials are urgently needed (75). In addition, a further literature review (76) reported that there is limited *in vitro* evidence on the efficacy of this drug against SARS-COV-2 and that clinical data based on studies with small sample size and affected by methodological limitations (77, 78). Therefore, high quality randomized clinical trials are strongly needed. Umifenovir interferes instead with the attachment of viral envelope protein to host cells (26). Umifenovir is an antiviral agent actually authorized in Russia, but not in Europe, for the treatment of Influenza A and B. This drug is considered safe and it is patented for the SARS treatment (79). The opinion of the Italian Medicine Agency on this drug is that evidence on its efficacy are not sufficient to support its use in patients with COVID-19 (80). Currently, a randomized, open label, parallel assignment clinical study is evaluating the efficacy and safety of umifenovir for the treatment of pneumonia in patients infected with SARS-COV-2 (NCT04260594). In this study, patients will be randomized to receive umifenovir plus basic treatment or just the basic treatment (Table 1). Moreover, two clinical trials are ongoing to assess the efficacy and safety of umifenovir and lopinavir/ritonavir (NCT04252885) or umifenovir, oseltamivir, and lopinavir/ritonavir (NCT04255017). Specifically, the NCT04252885 is a randomized, open label, parallel assignment clinical trial that will randomize patients with SARS-COV-2 infection in three groups (2:2:1). One group will receive the standard treatment plus lopinavir/ritonavir; the second group will receive standard treatment plus umifenovir; finally, the third group will just receive the standard treatment. The NCT04255017 is instead a randomized, single mask (participants), parallel assignment clinical trial that will randomize COVID-19 patients in four arms. One arm will receive the treatment with umifenovir; the second arm will receive the treatment with oseltamivir; the third arm will receive the treatment with lopinavir/ritonavir; the last arm will just receive the symptomatic supportive treatment (Table 1). Another small, randomized, triple mask (Participant, Care Provider, Investigator), parallel assignment clinical trial will be conducted on patients who have a positive test confirming COVID-19 to evaluate the combined treatment with umifenovir, interferon-β 1a, lopinavir/ritonavir, single dose of hydroxychloroquine, and the standards of care compared to the same combined treatment without umifenovir (NCT04350684).

**Table 2:** Mechanism of action, main adverse events and potential drug-drug interactions of inhibitors of viral invasion interfering with the S protein/ACE2 interaction, RAS inhibitors, and analogous ACE2 and A1-7 under clinical evaluation for the treatment of COVID-19.

**Therapeutic class**	**Drugs**	**Main mechanism of action**	**Main adverse events**	**Drug-drug interactions**	**References**
Inhibitors of S protein/ACE2 interaction	*Chloroquine/Hydroxychloroquine*	Increase of endosomal pH and interference with ACE2 glycosylation	Cardiovascular disorders, including prolongation of QT	Digoxin, class IA and III antiarrhythmic, tricyclic antidepressants, antipsychotics	(61, 62)
	*Umifenovir*	Interference with the attachment of the viral protein to host cells	Gastrointestinal symptoms and increased transaminase	As UDP-glucuronosyltransferase 1A9 and 2B7 inhibitor, umifenovir can increase levels of its substrates (paracetamol, buprenorphine, etc.) Cytochrome 3A4 inducers can reduce umifenovir levels	(63, 64)
ARBs	*Losartan*	Blocks the AII-induced lung injury	Dizziness, anemia, renal failure, asthenia, hyperkaliemia	Fluconazole and Rifampicine can increase losartan levels, Potassium-sparing diuretics can increase the risk of hyperkaelemia	(65, 66)
Analogous of ACE2 and A1-7	*A1-7*	Restores the beneficial effect of the non-classic RAS	Headache, fatigue, injection site reaction	Not Available	(29, 67, 68)
	*ACE2*	Restores the beneficial effect of the non-classic RAS	Hypernatremia, rash, dysphagia, and pneumonia	Not Available	(69)

In addition, speculations were done on the possible use for COVID-19 of new compounds, never approved before, which have shown the ability of interfering with S protein/ACE2 interaction (74). The compound SSAA09E2 showed the ability of blocking the early interaction of SARS-S protein with ACE2 in ACE2-expressing 293T cells (81). Moreover, the agent VE607 also showed a significant inhibition of SARS-pseudovirus entry in the same cellular model (82).

## New Pharmacological Perspective for COVID-19 Acting on the RAS

Based on the beneficial role of the non-classic RAS, which seems lacking in patients with COVID-19, hypotheses have been made on the potential therapeutic approach of restoring the ACE2/A1-7 pathway. This hypothesis is based on preclinical evidence showing an improvement of oxygenation, reduction of inflammation, and reduction of tissue fibrosis after infusion of A1-7 in two models of ARDS (65, 83). Evidence also showed that the administration of the soluble human recombinant ACE2 was able to reverse the lung-injury process in preclinical models of other viral infections (84, 85). The rationale to administer soluble ACE2 is to stimulate the RAS protective pathway without increasing the ACE2 transmembrane form that could instead potentiate the viral entry into the cells. Clinical evidence on this aspect is scarce (86). A phase 2 trial conducted in patients with ARDS showed that ACE2 infusion safely reduced the AII level, but this trial was not powered enough to show efficacy in terms of pulmonary function (69). Restoring the ACE2 activity may also be beneficial for the myocardial protection in patients with COVID-19 (87). To date, clinical researches are ongoing to assess the clinical impact of a restoration of the non-classic RAS (ACE2 and A1-7) in patients with COVID-19. Is underway a controlled trial aimed to assess the efficacy, safety and clinical impact of A1-7 infusion in a cohort of COVID-19 patients requiring mechanical ventilation (NCT04332666). It was, instead, suspended a further clinical trial that aimed to assess preliminary biologic, physiologic, and clinical data with the use of ACE2 recombinant compared to the standard care in patients with COVID-19 (NCT04287686).

In addition, based on the organ protective effects of RAS inhibitors, many studies are being conducted to investigate their efficacy in COVID-19 patients. The beneficial effects of ACE inhibitors and ARB may be related to the prevalence of ACE2/A1-7 effects as demonstrated in experimental studies (88, 89). Moreover, experimental evidence strongly suggests that AII could promote acute lung injury induced by different coronaviruses, including SARS-COV-1 and SARS-COV-2 (42, 65). Therefore, the use of RAS inhibitors may block the deleterious effect associated with AII. Two trials are ongoing to investigate the role of losartan for the treatment of COVID-19 in patients who have not previously received a RAS inhibitor and are either hospitalized (NCT04312009) or not hospitalized (NCT04311177). In particular, both trails (NCT04312009 and NCT04311177) are randomized, quadruple mask (participant, care provider, investigator, outcomes assessor), parallel assignment clinical trials that will compare the treatment with losartan vs. placebo in COVID-19 patients, including those with ARDS. Moreover, a pragmatic adaptive, randomized, quadruple mask (participant, care provider, investigator, outcomes assessor), parallel assignment trial is comparing the treatment with lopinavir/ritonavir, or hydroxychloroquine, or losartan vs. placebo in patients with COVID-19 (NCT04328012). Another randomized, quadruple mask (participant, care provider, investigator, outcomes assessor), parallel assignment clinical trial will evaluate the treatment with valsartan compared to placebo for the prevention of ARDS in hospitalized patients with COVID-19 (NCT04335786). Finally, a pilot, randomized, triple mask (participant, care provider, investigator), parallel assignment clinical trial is ongoing to assess the safety and efficacy of telmisartan compared to placebo for the mitigation of pulmonary and cardiac complications in COVID-19 patients (NCT04360551). Characteristics of the mentioned clinical trials are showed in Table 1. The mechanism of action, main adverse events and potential drug-drug interactions of RAS inhibitors and analogous of A1-7 and ACE2 under clinical evaluation for COVID-19 are summarized in Table 1.

Finally, other compounds that may be useful for the treatment of COVID-19, but not currently evaluated, are molecules that may adjust the imbalance between AT1 and AT2 receptors such as compound 21 (C-21), CGP-42112A, and L-163491 (26). C-21 and CGP-42112A are two agonists of AT2 receptors, whereas L-163491 has a dual action as a partial agonist of AT2 receptors and a partial antagonist of AT1 receptors (26).

## Conclusion

The RAS may play a complex role in SARS-COV-2 infection. SARS-COV-2 internalization may cause a reduction of ACE2 on cell surface. A reduction in ACE2 can further contribute to the pulmonary function deterioration and the myocardial damage. However, there is a paucity of clinical evidence on the efficacy of restoring the ACE2 functionality for the treatment of viral-induced lung injury. A clinical trial is ongoing to evaluate the effect of A1-7 in COVID-19 patients. To date, there is no effective drug for the treatment of COVID-19 and few clinical data are available. Some clinical trials are ongoing to evaluate the efficacy of drugs that could interfere with the S protein/ACE2 interaction such as umifenovir and hydroxychloroquine/chloroquine.

Data instead on the increased mRNA expression and levels of ACE2 after treatment with RAS inhibitors are scarce and to date not associated with an increased mortality in patients with COVID-19. Currently, clinical trials are ongoing to investigate the use of a RAS inhibitor for the reduction of the lung damage in patients with COVID-19. Substantial evidence is needed to guide decision-making on the use of ACE inhibitors and ARBs in such patients, until then we need to base on the available data that place RAS inhibitors among the safe choices for cardiovascular diseases.

## Author Contributions

AM, CS, CR, CF, GR, LB, GP, FR, and AC: drafting the work, revising it for important intellectual content, final approval of the version to be published, and agreement to be accountable for all aspects of the work in ensuring that questions related to the accuracy or integrity of any part of the work are appropriately discussed. FR and AC developed the concept and designed the study. AM wrote the paper. All authors contributed to the article and approved the submitted version.

## Conflict of Interest

The authors declare that the research was conducted in the absence of any commercial or financial relationships that could be construed as a potential conflict of interest.
